# Effects of Coordination and Strength Training on the Lower Extremity Inter-Segmental Coordination of Instep Kicking

**DOI:** 10.3390/bioengineering13010019

**Published:** 2025-12-25

**Authors:** Liwen Zhang, Meizhen Zhang, Hui Liu

**Affiliations:** 1 Biomechanics Laboratory, School of Physical Education and Health Engineering, Taiyuan University of Technology, Taiyuan 030024, China; zhangliwen1399@163.com (L.Z.); meizhen1116@163.com (M.Z.); 2 Biomechanics Laboratory, College of Human Movement Science, Beijing Sport University, Beijing 100084, China

**Keywords:** motor control, dynamic system theory, coupling angle, training effect, kicking performance improvement

## Abstract

The purpose of this study was to determine the effects of coordination training and strength training on the lower extremity inter-segmental coordination during instep kicking for novices. Thirty-two male college students with no soccer-specific training experience participated and were randomly assigned to either a coordination training group, a strength training group, or a kicking training group. Both the coordination and strength training groups also performed the same kicking training as the kicking training group. Each participant executed exercise training three times a week for eight weeks. The instep kicking test was performed before and after the three training sessions. Two-way ANOVAs were conducted to determine the training effects on the kicking performance and the inter-segmental coordination. The maximum ball speed significantly increased for all three training groups (*p* < 0.001, effect size = 0.638). In contrast, improvements in kicking accuracy were specific to the coordination training group (*p* = 0.001, effect size = 0.326), with no significant changes observed in the strength (*p* = 0.052, effect size = 0.138) or kicking groups (*p* = 0.953, effect size < 0.001). The time spent percentage of the knee-ankle shank-phase coordination pattern in the leg-cocking phase was significantly increased (*p* = 0.003, effect size = 0.268), but the time spent percentage of the hip-knee thigh-phase in the back swing phase significantly decreased after the three trainings (*p* = 0.031, effect size = 0.150). A significant reduction in the relative activity of the tibialis anterior and gastrocnemius muscles occurred exclusively after coordination training (*p* = 0.024, effect size = 0.188). This study confirms that coordination training provides a unique contribution to skill acquisition in novices, specifically enhancing kicking accuracy and neuromuscular control, whereas improvements in maximal ball speed were generic to all training types.

## 1. Introduction

Inter-segmental coordination is one of the key factors influencing techniques and performance in sports [1,2]. Inter-segmental coordination refers to the mastering of redundant degrees of freedom of the musculoskeletal system employed during sport movements to produce a controllable system [3], and could be described using the vector coding method that calculates the angle of the vector between two adjacent data points in time on the angle-angle diagram relative to the right horizontal, defined as a coupling angle ranged from 0° to 360° [4]. In addition, the relative activity between agonist and antagonist muscles, defined as the ratio of the two muscles, describes the coordinated movement of the muscles during a specific sport movement [5]. It has been suggested that athletes achieving better sports performance have more appropriate characteristics of the inter-segmental coordination in running [6], kicking [7,8,9], swimming [10,11], ice climbing [12], and ski jumping [13].

Instep kicking is one of the most fundamental and frequently used skills in the sport of soccer. Improving the inter-segmental coordination of instep kicking is critical for achieving a high ball speed and improving the chances of scoring in soccer [1,2]. Several previous studies have found that experienced soccer athletes had better control of the distal joints compared to novices [7,9,14,15]. Our recent study further revealed that compared to the novice, the experienced soccer athletes had more knee flexion dominant coordination patterns in the back swing and leg-cocking phases and knee extension dominant patterns in the leg acceleration phase of the instep kicking movement, which contribute significantly to the increased ball speed [1]. We also found that the experienced soccer athletes had lower relative activity of the tibialis anterior and gastrocnemius muscles, which increases the kicking accuracy [1]. These results from the previous literature [1,2,7,9,14,15] specified the relationships between the inter-segmental coordination and instep kicking performance, and provided evidence for the evaluation of training effects in soccer. Critically, the variations in inter-segmental coordination observed between experts and novices indicate that such coordinative attributes are malleable and can be improved through targeted training approaches. This evidence provides a foundational rationale for evaluating the effects of specific training interventions on neuromuscular coordination in novice populations.

The coordination training and strength training that are widely used in soccer practice may theoretically improve the inter-segmental coordination during instep kicking by improving the motor control ability of the central nervous system [16,17,18,19]. The specific effects of the coordination training and strength training on the inter-segmental coordination in instep kicking, however, is unclear. The previous literature [20] has reported that, although chronic soccer coordination training is beneficial to develop interlimb synchronization capabilities examined by the movement of the hand and foot under various directions and frequencies, the counter movement jump height proved to be a good predictor of the hand and foot coordinated movement in the in-phase and anti-phase modes, explaining approximately 30% of the variance. Another longitudinal study reported that the shuttle test with direction and sprint time changes decreased significantly both after a short period of coordination training and strength training, but more so in the reference and coordination training groups compared to the strength training group [21]. The reasons for such multiple results from the previous literature may be related to the choice of indicators. Although the various shuttle tests with changes in direction and the hand and foot coordinated movement could provide a variety of information about the motor control capability of the nervous system, the information about the dynamic coupling among lower extremity segments and muscles from a dynamical systems perspective of motor control in the kicking movement is still unclear.

The purpose of this study was to determine the effects of coordination training and strength training on the lower extremity inter-segmental coordination during instep kicking for novices. We hypothesized that eight weeks of coordination training or strength training would significantly increase the time spent percentage of knee flexion dominant coordination patterns in the back swing and leg-cocking phases and knee extension dominant patterns in the leg acceleration phase of the instep kicking movement, and decrease the relative activity of the tibialis anterior and gastrocnemius muscles.

## 2. Materials and Methods

### 2.1. Participants

A total of 32 healthy male college students with no soccer-specific training experience and right foot dominance volunteered to participate in this study. This study focused on male participants to maintain consistency with previous biomechanical studies of soccer kicking [1,2,8], while also controlling for potential sex-based differences in neuromuscular adaptation. A priori sample size calculation was performed using G*Power software (version 3.1.9.4; Heinrich-Heine-Universität Düsseldorf, Düsseldorf, Germany). For a repeated-measures analysis of variance (ANOVA) with within-between interaction, assuming an effect size f of 0.4, an α error probability of 0.05, a power (1-β) of 0.8, 3 groups, and 2 measurements (pre- and post-test); the calculation indicated a total sample size of 27 participants (9 per group) would be required. The assumed large effect size (f = 0.4) was selected based on previous studies reporting substantial training-induced adaptations in athletes [22,23]. To compensate for potential attrition, 36 healthy male college students (12 per group) were initially recruited. Ultimately, 32 participants completed the entire study protocol. Participants were randomly assigned to either a coordination training group, a strength training group, or a kicking training group (Table 1). One-way ANOVA revealed no significant differences in age, height, or body weight at baseline among the three groups (Table 1). The use of human subjects was approved by the Institutional Review Board of Beijing Sport University. Written consent was obtained from each participant before any data collection.

### 2.2. Procedures

Each participant completed a pre-training test, followed by an eight-week training intervention specific to their group assignment: coordination plus kicking training (coordination group), strength plus kicking training (strength group), or kicking-only training (control group). All participants then underwent a post-training test. This parallel-group design allows us to isolate the specific effects of coordination and strength training over and above the foundational learning achieved through standard football practice. In each of the pre- and post-training tests, the participants completed an instep kicking test to collect lower extremity three-dimensional (3-D) kinematic and electromyographic (EMG) data for the dominant leg.

#### 2.2.1. Kicking Test

**Figure 1:**
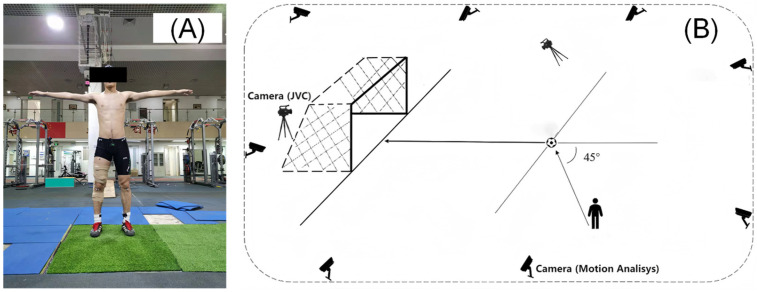
Experimental setup for the instep kicking test, including the schematic of marker and EMG electrode placement on the subject (**A**) and the schematic of the testing area (**B**).

The kicking test was conducted to assess the inter-segmental coordination and performance outcomes (ball speed and accuracy) of the instep kick before and after the training interventions. A warm-up consisting of 10 min of running, stretching exercises for the lower limbs, and several practice kicks was conducted. A total of 19 passive reflective markers were placed bilaterally on each participant’s lower extremity (Figure 1A), including the anterior superior iliac spine (ASIS), mid-thigh, medial and lateral femoral condyles, tibial tuberosity, medial and lateral malleoli, posterior calcaneus, and first and fifth metatarsal heads. An additional marker was attached at the junction of the 4th and 5th lumbar spine vertebra (L4-5). A reflective marker was also attached to the soccer ball for estimating the time of foot–ball contact. Surface EMG electrodes were placed over the muscle belly of the rectus femoris, lateral femoris, medial femoris, semimembranosus, biceps femoris, tibialis anterior, and lateral gastrocnemius muscle of the dominant leg (Figure 1A). A maximal voluntary contraction (MVC) was performed to collect MVC EMG data.

The markers on the medial femur condyles and malleoli were removed after a standing calibration. Each participant then performed ten successful trials for instep kicking tasks after an approach of two steps angled to 45° to the kicking direction. Kicking trials were performed against a goalpost (3 height × 2 m width) located five meters from the ball. A standard-sized and inflated ball was used for measurements. A one-minute rest interval between consecutive kicking trials was provided. Considering the speed and accuracy demand, the participant was instructed to kick the ball against the center of the goal as hard as possible.

The 3-D trajectories of the reflective markers were recorded using a Motion Analysis videographic data acquisition system (Raptor-4; Motion Analysis Inc., Santa Rosa, CA, USA) with 8 cameras at a sample rate of 200 frames/s (Figure 1B). EMG data were recorded using a Delsys Trigno™ Wireless EMG acquisition system (Delsys Inc., Natick, MA, USA) at a sample rate of 2000 samples/channel/s. The videographic and EMG data collections were time synchronized using the Delsys Trigger Synchronizer (Delsys Inc., Natick, MA, USA).

Two digital video cameras (GC-PX100, JVC, Tokyo, Japan) with a resolution of 1920 × 1080 at a sampling frequency of 60 frames/s were used to record the 2-D coordinates of the ball trajectory and the position where the football enters the goal, respectively. One camera was set up five meters to the right side of the ball and perpendicular to the sagittal plane of the kicking movement. Another camera was set up behind the goal and parallel to the sagittal plane of the kicking movement (Figure 1B).

#### 2.2.2. Interventions

Participants in each experimental group had three intervention sessions per week for eight weeks, with at least 24 h between sessions. Participants in the kicking training group had 5 min of warm-up, 15 min of standardized instep kicking training drills, and 5 min of post-training cool-down (Table 2). Participants in the coordination training group had 30 min of various forms of ladder, running, and jumping drills, while participants in the strength training group had 30 min of resistance training drills for the lower extremities (Table 2). The coordination training, centered on agility ladder drills was designed to enhance fundamental coordinative capacities such as dynamic balance, inter-limb coordination, and movement efficiency in multi-directional patterns [19]. These capacities are theorized to form the basis for improving the inter-segmental coordination of complex soccer-specific skills like the instep kick. The 8-week strength training protocol was adapted from [24,25], which has been demonstrated to significantly improve the maximal strength (e.g., the peak torque of the hip extensors, knee extensors, and ankle dorsiflexors), soccer kick movement pattern, and ball speed. Furthermore, the participants in the coordination and strength training group had the same instep kicking training drills as the kicking training group (Table 2).

### 2.3. Data Reduction

A kicking movement cycle was defined as the duration from toe-off of the kicking leg to the contact of the kicking foot with the ball and was divided into three phases. The back swing phase begins with the toe-off of the kicking leg and ends with maximum hip extension of the kicking leg. The leg-cocking phase begins with maximum hip extension of the kicking leg and ends with maximum knee flexion of the kicking leg. The leg acceleration phase begins with maximum knee flexion of the kicking leg and ends with contact of the kicking foot with the ball.

The raw 3-D trajectories of all but one reflective markers were filtered using a Butterworth low-pass filter at an estimated optimum frequency of 13 Hz [26]. The raw 3-D trajectories of the single marker on the ball were not filtered, as any filtering of this trajectory created ambiguity in the identification of the specific frame when ball impact occurred [27]. The pelvis, thigh, shank, and foot reference frames were defined as previously described [28]. The hip, knee, and ankle Cardan joint angles between adjacent segment reference frames were calculated in an order of flexion–extension, valgus–varus, and internal rotation [28], and were normalized and time-scaled to 100% of the kicking movement.

The vector coding method was used to quantify the hip-knee and knee-ankle coordination patterns of the kicking leg over the time course of kicking. The coupling angle (γ) for each time interval was derived as the angle from the horizontal of a vector connecting two consecutive data points on the angle-angle plot of the hip-knee and the knee-ankle motion of the kicking leg [4]:

$$\gamma_{j,i}=\tan^{-1}\left(\frac{y_{j,i+1}-y_{j,i}}{x_{j,i+1}-x_{j,i}}\right)$$

where 0° ≤ γ ≤ 360°, and i is a data point of the jth trial. Mean coupling angle $\;({\bar{\gamma }}_{i})$ were calculated based on the average horizontal and vertical components at each instant using circular statistics due to the directional nature of coupling angle [29]. Within a subject and then across the group, ${\bar{\gamma }}_{i}$ was calculated from the mean horizontal (${\bar{x}}_{i}$) and vertical (${\bar{y}}_{i}$) components at each data point:

$${\bar{x}}_{i}=\frac{1}{n}\sum_{j=1}^{n}\left(\cos y_{j,i}\right)$$

$${\bar{y}}_{i}=\frac{1}{n}\sum_{j=1}^{n}\left(\sin y_{j,i}\right)$$

$${\bar{\gamma }}_{i}=\left\{\begin{array}{c} \arctan\left({\bar{y}}_{i}/{\bar{x}}_{i}\right),\;if\;{\bar{x}}_{i}>0\\ 180+\arctan({\bar{y}}_{i}/{\bar{x}}_{i}),\;if\;{\bar{x}}_{i}<0 \end{array}\right.$$

Based on the mean coupling angle, four coordination patterns were defined (Table 3), including in-phase (rotating in the same direction), anti-phase (rotating in the opposite direction), proximal-phase (the hip is rotating dominantly in hip-knee coupling, and the knee is rotating dominantly in the knee-ankle coupling), and distal-phase (the knee is rotating dominantly in hip-knee coupling, and the ankle is rotating dominantly in the knee-ankle coupling). The time spent percentage of each coordination pattern in each kicking phase was calculated.

The raw EMG signals were filtered using a band-pass digital filter at a low-pass cutoff frequency of 800 Hz and a high-pass cutoff frequency of 10 Hz, and then rectified [30]. The band-pass filtered and rectified EMGs were filtered using a low-pass digital filter again at a cutoff frequency of 20 Hz to obtain the linear envelop EMGs [30]. Subsequently, the EMG amplitude for each muscle was normalized to its corresponding MVC value obtained during the pre-test maximal contractions. To enhance the robustness of the relative activity indices against noise and crosstalk, electrode placement followed established anatomical guidelines [31]. The relative activity between agonist and antagonist muscles was calculated by the ratio of the envelop EMGs of the quadriceps femoris and hamstrings, tibialis anterior and gastrocnemius muscles, respectively [5]. Average relative activity between agonist and antagonist muscles was then calculated.

The 2-D coordinates of the ball center in the video were obtained using Fastmove 3-D Motion (FastMove Inc., Dalian, China). The maximum ball speed was defined as the maximum value of the ratio of the displacement of the ball to the time between every two adjacent frames. The kicking accuracy was defined as the distance between the ball center and the goal center at the moment when the ball enters the goal, which was obtained by the video shooting. While it is acknowledged that kicking accuracy in a real-game context is influenced by additional factors, this metric was chosen for its objectivity, reliability, and widespread use in biomechanical studies [32].

### 2.4. Data Analysis

Two-way ANOVAs were performed to compare the maximal ball speed, the distance between the ball center and the goal center when the ball enters the goal, the time spent percentage of each of the hip-knee and knee-ankle coordination patterns, and the averaged relative activity between agonist and antagonist muscles between the three groups pre- and post-training period. Paired *t*-tests were performed as post hoc tests to locate differences if no significant interaction of independent variables was detected, but significant main effects were detected. One-way ANOVAs were performed to determine the effects of each independent variable on a given dependent variable if a significant interaction effect of independent variables was detected. Effect sizes (ES) were reported as partial eta squared (η^2^p) for ANOVAs and Hedge’s g for *t*-tests. Given the slight unevenness in group sizes, the results were further verified using a Generalized Linear Model (GLM), which yielded consistent findings and confirmed the robustness of the conclusions. All post hoc comparisons were adjusted using the Bonferroni correction for multiple comparisons. A type I error rate greater than or equal to 0.050 was chosen as the indication of statistical significance.

## 3. Results

No significant interaction effect of training group and time was detected on the maximum ball speed (Table 4). A significant main effect of training time on the maximum ball speed was detected. Paired *t*-tests revealed that the maximum ball speed in the three groups was significantly increased in the post-training test compared to the pre-training test. A significant interaction effect of group and time on the distance between ball center and goal center was detected (Table 4). One-way ANOVAs revealed that the distance between ball center and goal center in the coordination training group was significantly decreased in the post-training test compared to the pre-training test (*p* = 0.001, ES = 0.326).

No significant interaction effect of training group and time was detected on the time spent percentage of hip-knee in-phase, thigh-phase, and shank-phase coordination patterns in the back swing phase (Table 5). A significant main effect of training time on the time spent percentage of the hip-knee thigh-phase coordination patterns was detected. Paired *t*-tests revealed that the time spent percentage of hip-knee thigh-phase coordination patterns in the three groups was significantly decreased in the post-training test compared to the pre-training test. A significant main effect of group on the time spent percentage of hip-knee in-phase and shank-phase coordination patterns was detected. Paired *t*-tests revealed that the time spent percentage of the hip-knee in-phase coordination pattern (*p* = 0.013) and strength (*p* = 0.023) training groups was significantly lower than that of the kicking training group, both in pre- and post-training tests. Paired *t*-tests also revealed that the time spent percentage of the hip-knee shank-phase coordination pattern of the coordination training group was significantly greater than that of the kicking training group, both in pre- and post-training tests (*p* = 0.013). A significant interaction effect of group and time on the time spent percentage of the hip-knee anti-phase coordination pattern in the back swing phase was detected. One-way ANOVAs revealed that the time spent percentage of the hip-knee anti-phase coordination pattern in the coordination training group was significantly increased in the post-training test compared to the pre-training test (*p* < 0.001).

No significant interaction effect of training group and time was detected on the time spent percentage of each knee-ankle coordination pattern in the back swing phase (Table 5). A significant main effect of training time on the time spent percentage of the knee-ankle in-phase coordination pattern was detected. Paired *t*-tests revealed that the time spent percentage of the knee-ankle in-phase coordination pattern in all groups was significantly increased in the post-training test compared to the pre-training test.

No significant interaction effect of training group and time was detected on the time spent percentage of each hip-knee coordination pattern in the leg-cocking phase (Table 6). A significant main effect of training time on the time spent percentage of the hip-knee in-phase coordination pattern was detected. Paired *t*-tests revealed that the time spent percentage of the hip-knee in-phase coordination pattern in the three groups was significantly decreased in the post-training test compared to the pre-training test.

No significant interaction effect of training group and time was detected on the time spent percentage of each knee-ankle coordination pattern in the leg-cocking phase (Table 6). A significant main effect of training time on the time spent percentage of the knee-ankle anti-phase and shank-phase coordination pattern was detected. Paired *t*-tests revealed that the time spent percentage of knee-ankle anti-phase coordination pattern in the three groups was significantly decreased in the post-training test compared to the pre-training test, while the time spent percentage of knee-ankle shank-phase coordination pattern in the three groups was significantly increased in the post-training test compared to the pre-training test.

No significant interaction effect of training group and time was detected on the time spent percentage of each hip-knee and knee-ankle coordination patterns in the leg acceleration phase (Table 7). No significant main effects of group and time were detected on the time spent percentage of each hip-knee and knee-ankle coordination pattern (Table 7).

No significant interaction effect of training group and time was detected on the relative activity of the quadriceps femoris and hamstrings in the kicking movement (Table 8). No significant main effects of group and time were detected on the relative activity of the quadriceps femoris and hamstrings. A significant interaction effect of group and time on the relative activity of the tibialis anterior and gastrocnemius in the kicking movement was detected (Table 8). One-way ANOVAs revealed that the relative activity of the tibialis anterior and gastrocnemius in the coordination training group was significantly decreased in the post-training test compared to the pre-training test (*p* = 0.024, ES = 0.188). Paired *t*-tests revealed that there was no significant difference in the relative activity of the tibialis anterior and gastrocnemius between the three groups in the pre-training test, but the relative activity of the tibialis anterior and gastrocnemius of the coordination training group (*p* = 0.006) and strength training group (*p* = 0.021) was significantly lower than that of the kicking training group in the post-training test (Table 8).

## 4. Discussion

The results of this study partially support our hypothesis that eight weeks of coordination training or strength training would significantly increase the time spent percentage of knee flexion dominant coordination patterns in the back swing and leg-cocking phases and knee extension dominant patterns in the leg acceleration phase of the instep kicking movement, and decrease the relative activity of the tibialis anterior and gastrocnemius muscles. The results showed that in the back swing phase of the kicking movement, the time spent percentages of hip-knee thigh-phase coordination patterns were significantly decreased. While in the leg-cocking phase of the kicking movement, the time spent percentages of the hip-knee in-phase coordination pattern and knee-ankle anti-phase were significantly decreased, but the time spent percentage of the knee-ankle shank-phase was significantly increased after the eight weeks of single kicking training, or kicking training combined with coordination training or strength training. Further, the time spent percentages of each of the hip-knee and knee-ankle coordination patterns in the leg acceleration phase were not significantly changed after the eight weeks of training. Our recent study demonstrated that compared with the novices, the experienced athletes achieved a higher maximum ball speed and had less hip-knee thigh-phase coordination patterns in the back swing phase, as well as less hip-knee in-phase, knee-ankle anti-phase, and more knee-ankle shank-phase coordination patterns in the leg-cocking phase during the kicking movement [1]. Our recent study also demonstrated that the maximum ball speed was significantly negatively correlated with the time spent percentage of hip-knee thigh-phase coordination patterns in the back swing and knee-ankle anti-phase coordination patterns in the leg-cocking phase of the kicking movement [1]. These results taken together indicated that the lower extremity inter-segmental coordination of the kicking leg was significantly improved after eight weeks of single kicking training, or kicking training combined with coordination training or strength training. The observed shifts in coordination patterns, particularly the reduced reliance on proximal segments, indicate an adaptation toward more efficient motor control strategies, consistent with the early stages of skill acquisition. Specifically, these results indicated that regardless of the training programs used, more knee flexion dominant coordination patterns in the back swing and leg-cocking phases were involved, but no more knee extension dominant coordination patterns in the leg acceleration phase were involved after training.

The results of the present study were consistent with the literature. Previous studies had shown that there was an increasement in the knee flexion before the hip flexion after 10 weeks of instep kicking practice for novices, and this flexion continued to occur as the hip was flexed forward [7], suggesting a reduced dynamic involvement of the proximal segment (hip) but with increased involvement of the distal segments (knee) after instep kicking practice [7,9]. These findings lend some support to Bernstein’s ideas on the acquisition of skilled behavior [3]. Bernstein originally argued that the acquisition of skill progresses from a reorganization of motor system degrees of freedom and suggested that a learner initially demonstrates rigid and awkward coordination modes, subsequently known as freezing joint motions, to cope with the abundance of motor system degrees of freedom [3]. As control over degrees of freedom is gained, joint motion is gradually released or freed [3]. The results of this study, combined with the results of previous studies, indicated that the knee joint was given greater freedom of movement as the freezing constraints were lifted after a period of training, and a coordinative structure that enabled the knee joint to take greater advantage of the flexion movement in the back swing and leg-cocking phases of the kicking movement developed. The greater freedom of movement for the knee flexion further contributed to increasing the knee extension torque before foot–ball contact and the final ball speed by stretching the knee extensor muscles at the beginning of the kicking movement [8,33].

Although the lower extremity inter-segmental coordination was improved after the eight weeks of single kicking training, or kicking training combined with coordination training or strength training, suggesting a beneficial effect of general practice, the results of the present study indicated that the coordination training had distinct positive effects on the improvement of inter-segmental coordination during instep kicking as evidenced by the unique improvements in specific coordinative aspects. On the one hand, the results showed that only after the coordination training did the distance between the ball center and the goal center when the ball enters the goal decrease, which means that the kicking accuracy improved. The results also showed that only after the coordination training did the relative activity of the tibialis anterior and gastrocnemius muscles decrease. Our recent study demonstrated that the relative activity of the tibialis anterior and gastrocnemius muscles of experienced athletes was significantly lower than that of novices during the instep kicking movement [1]. We also found that the lower relative activity of the tibialis anterior and gastrocnemius muscles, the better the kicking accuracy [1]. The coordination training programs used in this study primarily incorporated ladder drills, complemented by directional running and jumping exercises. The ladder drills could enhance the dynamic coupling among lower extremity muscles by alerting the foot speed and direction rapidly across multiple planes, which was one of the most important aspects for faster and more accurate shooting during the instep kicking movement [34]. These results taken together suggest a strong link between coordination training and improved kicking accuracy, likely mediated by the enhanced coordinated activation of lower extremity muscles. While potential confounding factors exist, the specific and concurrent improvements in both outcome (accuracy) and mechanism (relative activity) exclusively in the coordination group strengthen this interpretation.

On the other hand, the results showed that only after the coordination training did the time spent percentage of the hip-knee anti-phase coordination pattern in the back swing phase increase. This was supported by the results of our recent study that demonstrated a greater time spent percentage of hip-knee anti-phase coordination pattern in the back swing phase for the experienced soccer athletes compared to the novices [1]. Similarly to the theory of Bernstein, studies of inter-limb coordination during oscillation on a ski simulator [35], volley ball serves [36], or swimming [11] showed that beginners freeze the degrees of freedom while experts release the degrees of freedom not useful to the task. Freezing the degrees of freedom is mostly related to a basic coordination mode, like the in-phase [37], while releasing the degrees of freedom corresponds to the anti-phase coordination mode [11,35,36]. Another previous study indicated that the in-phase coordination pattern in human movement emerges spontaneously without requiring additional neural input from the brain, whereas the anti-phase coordination pattern necessitates more direct and deliberate neural control [38]. The findings of this study suggest that coordination training may enhance specific aspects of neuromuscular and motor control functions underlying the instep kicking movement by facilitating a shift toward more complex coordination modes.

The strength training did not have an additional effect on the improvement of inter-segmental coordination compared with the single kicking training or kicking training combined with coordination training in the present study. One possible explanation for the lack of effect is that our strength intervention was not long enough in duration to induce the altered relative activity pattern of the lower extremity muscles. It has been suggested that kicking performance was achieved through an altered relative activity pattern after strength training [25]. The participants in the strength training group might still have been in an early stage of training, and their increase in the maximum ball speed might be mainly due to increased muscle strength without significantly altered muscle relative activity. Another likely explanation is that the type of strength training programs used in this study was resistance training consisting of low-intensity and multiple repetitions exercises, which may be less effective in altering relative muscle activity compared to the explosive muscle strength training programs. Future studies are needed to determine if more explosive or sport-specific strength training can elicit different effects on coordination.

Although the current study evaluated the immediate effects of eight weeks of coordination training and strength training on the lower extremity inter-segmental coordination of instep kicking, further investigations are needed to confirm the effects of different training periods on inter-segmental coordination to understand the learning and development of motor skills from the perspective of motor control. Also, the coordination training program selected in this study mainly focuses on the common ladder training, supplemented by a mixed training program of various directional running and jumping movements. Additional studies are needed to evaluate the effect of separate ladder training, directional running training, jumping training, and other various coordination training programs, such as soccer game training, etc., on the inter-segmental coordination of instep kicking. It should be noted that the findings of this study are based on a specific cohort of young, male, untrained students, which may limit their generalizability to other populations, such as females or trained athletes. Future studies are needed to verify these effects in more diverse groups.

## 5. Conclusions

Maximum ball speed and lower extremity inter-segmental coordination were significantly improved after the eight weeks of single kicking training, or kicking training combined with coordination training or strength training. Specifically, more knee flexion dominant coordination patterns in the back swing and leg-cocking phases were involved, but no more knee extension dominant coordination patterns in the leg acceleration phase were involved after training. The findings suggest that the coordination training program was associated with significant improvements in kicking accuracy, accompanied by a reduction in the relative activity between tibialis anterior and gastrocnemius. These parallel changes indicate that the accuracy gains are likely mediated through enhanced neuromuscular coordination of the lower extremity. Strength training programs did not have an additional effect on the improvement of inter-segmental coordination compared with the single kicking training or kicking training combined with coordination training in this study. The coordination training elicited specific neuromuscular adaptations that were not observed following strength training, highlighting its unique role in enhancing movement precision during early-stage skill acquisition.

## Figures and Tables

**Table 1:** Descriptions of participants (mean ± SD).

	Training Group			*p* Value	η^2^p
	Coordination (*n* = 12)	Strength (*n* = 10)	Kicking (*n* = 10)		
Age (year)	21.5 ± 1.7	21.0 ± 1.8	21.7 ± 2.3	0.757	0.019
Body mass (kg)	74.0 ± 9.5	69.9 ± 9.5	68.3 ± 9.1	0.352	0.070
Standing height (cm)	178.2 ± 7.0	175.3 ± 5.5	177.0 ± 6.0	0.090	0.153

**Table 2:** Training exercises.

Training Group		Weeks		
		1–2	3–5	6–8
Coordination training group	Exercise	(1) Forward and backward line hops (2) Lateral line hops (3) Scissors (4) Two-cone drills forward run (5) Ladder drills (One in the hole, lateral two in the hole, ickey shuffle, two in two out)	(1) Forward and backward line hops single-leg variation (2) Lateral line hops single-leg variation (3) Traveling scissors (4) Three-cone 180-degree drill (5) Ladder drills (cha-cha, carioca, Billy Sims crossover, Hopscotch)	(1) Forward and backward line hops single-leg variation across obstacles (2) Lateral line hops single-leg variation across obstacles (3) 180-degree traveling line hop single-leg variation (4) Four-cone T drill (5) Ladder drills (Ali shuffle, Slaloms, cherry pickers, 180 s)
		Instep kicking training		
	Reps and sets	(1)–(3):10 reps × 2 sets, (4): 5 reps × 2 sets, (5): 10 s/drill × 3 sets		
	Rest	30 s between sets, 2 min between exercises		
Strength training group	Exercise	Hip abduction, hip adduction, knee extension, knee flexion, ankle plantar flexion, ankle dorsiflexion		
		Instep kicking training		
	Reps and sets	10 reps × 3 sets	10 reps × 3 sets	6 → 5 → 4 → 3 → 2 reps × 5 sets
	Load	50% of 1 RM	70% of 1 RM	70 → 75 → 80 → 85 → 90% of 1 RM
	Rest	60 s between sets, 3 min between exercises	60 s between sets, 3 min between exercises	90 s between sets, 3 min between exercises
Kicking training group	Exercise	Instep kicking training		

Abbreviations: reps = repetitions, RM = repetition maximum.

**Table 3:** Scheme used to categorize coordination patterns.

Coordination Pattern	Coupling Angle Definitions
In-phase	22.5°≤γ<67.5° , 202.5°≤γ<247.5°
Anti-phase	112.5°≤γ<157.5° , 292.5°≤γ<337.5°
Proximal-phase	0°≤γ<22.5° , 157.5°≤γ<202.5° , 337.5°≤γ<360°
Distal-phase	67.5°≤γ<112.5° , 7.5°≤γ<292.5°

**Table 4:** Comparison of maximum ball speed and the distance between the ball center and the goal center between training groups and periods.

Kicking Performance	Time	Training Group			Time × Group Effect		Group Effect		Time Effect	
		Coordination (*n* = 12)	Strength (*n* = 10)	Kicking (*n* = 10)	*p* Value	η^2^p	*p* Value	η^2^p	*p* Value	η^2^p
Maximum ball speed (m/s)	Pre-training	13.08 ± 2.54	13.71 ± 2.53	12.90 ± 2.19	0.689	0.025	0.873	0.009	<0.001	0.638
	Post-training	15.49 ± 3.09	15.56 ± 2.00	14.37 ± 1.29						
Distance between ball and goal center (m)	Pre-training	1.18 ± 0.29	1.02 ± 0.24	0.93 ± 0.23	0.047	0.082	0.270	0.096	0.004	0.483
	Post-training	0.83 ± 0.18	0.82 ± 0.27	0.89 ± 0.14						

**Table 5:** Comparison of the time spent percentage of each coordination pattern in the back swing phase between training groups and time.

Segment	Patterns	Time	Training Group			Time × Group Effect		Group Effect		Time Effect	
			Coordination (*n* = 12)	Strength (*n* = 10)	Kicking (*n* = 10)	*p* Value	η^2^p	*p* Value	η^2^p	*p* Value	η^2^p
Hip-knee	In- phase	Pre-training	38.0 ± 17.2 ^B^	30.0 ± 15.0 ^B^	44.0 ± 18.2 ^A^	0.106	0.143	**0.025**	0.224	0.368	0.028
		Post-training	29.7 ± 18.6 ^B^	39.2 ± 21.8 ^B^	54.0 ± 12.7 ^A^						
	Anti-phase	Pre-training	4.4 ± 2.6	7.9 ± 5.3	7.3 ± 5.8	**0.008**	0.285	0.980	0.001	**0.003**	0.261
		Post-training	10.9 ± 5.2	8.2 ± 7.4	8.4 ± 4.8						
	Thigh-phase	Pre-training	8.5 ± 6.1	13.6 ± 9.0	9.8 ± 5.8	0.337	0.072	0.323	0.075	**0.031**	0.150
		Post-training	6.7 ± 4.9	7.2 ± 2.5	8.1 ± 5.1						
	Shank-phase	Pre-training	49.1 ± 21.5 ^B^	48.6 ± 16.8	38.9 ± 17.9	0.309	0.078	**0.041**	0.197	0.252	0.045
		Post-training	52.7 ± 19.5 ^B^	41.1 ± 20.1	29.4 ± 10.7						
Knee-ankle	In- phase	Pre-training	24.9 ± 17.9	22.7 ± 15.9	37.3 ± 20.2	0.356	0.069	0.079	0.160	**0.012**	0.199
		Post-training	28.9 ± 16.7	40.0 ± 15.8	46.3 ± 23.1						
	Anti-phase	Pre-training	3.9 ± 4.2	8.2 ± 9.0	6.7 ± 6.7	0.501	0.047	0.419	0.058	0.091	0.156
		Post-training	6.6 ± 4.4	7.6 ± 10.1	4.9 ± 3.9						
	Shank-phase	Pre-training	57.8 ± 21.9	40.4 ± 26.0	39.1 ± 21.7	0.699	0.024	0.099	0.147	0.097	0.092
		Post-training	46.0 ± 24.0	32.7 ± 11.7	36.2 ± 22.6						
	Foot-phase	Pre-training	13.4 ± 10.4	28.6 ± 23.5	17.0 ± 12.7	0.200	0.105	0.189	0.108	0.405	0.024
		Post-training	18.5 ± 21.0	19.8 ± 8.6	12.6 ± 7.3						

^A^ indicates statistical significance compared to the strength training group. ^B^ indicates statistical significance compared to the kicking training group. The bold in *p*-Value indicates a significant difference (*p* < 0.05).

**Table 6:** Comparison of the time spent percentage of each coordination pattern in the leg-cocking phase between training groups and time.

Segment	Patterns	Time	Training Group			Time × Group Effect		Group Effect		Time Effect	
			Coordination (*n* = 12)	Strength (*n* = 10)	Kicking (*n* = 10)	*p* Value	η^2^p	*p* Value	η^2^p	*p* Value	η^2^p
Hip-knee	In- phase	Pre-training	0.5 ± 1.4	1.3 ± 1.3	1.1 ± 2.4	0.480	0.049	0.584	0.036	**0.009**	0.213
		Post-training	0.1 ± 0.5	0.1 ± 0.3	0.0 ± 0.1						
	Anti-phase	Pre-training	48.0 ± 12.2	45.9 ± 10.4	47.1 ± 10.0	0.327	0.074	0.450	0.054	0.822	0.002
		Post-training	51.3 ± 12.9	46.4 ± 15.4	41.7 ± 9.4						
	Thigh-phase	Pre-training	18.1 ± 4.4	15.9 ± 8.0	14.3 ± 3.7	0.790	0.016	0.118	0.137	0.934	<0.001
		Post-training	17.5 ± 3.0	15.6 ± 3.7	15.5 ± 3.1						
	Shank-phase	Pre-training	33.4 ± 12.9	36.9 ± 14.1	37.5 ± 10.2	0.420	0.058	0.255	0.090	0.822	0.002
		Post-training	31.1 ± 14.1	35.6 ± 16.9	42.8 ± 7.2						
Knee-ankle	In- phase	Pre-training	6.7 ± 4.6	7.0 ± 5.8	7.5 ± 8.0	0.808	0.015	0.930	0.005	0.071	0.108
		Post-training	4.4 ± 7.7	4.9 ± 6.0	3.1 ± 2.0						
	Anti-phase	Pre-training	33.0 ± 21.3	41.1 ± 17.7	32.7 ± 18.6	0.328	0.074	0.485	0.049	**0.039**	0.138
		Post-training	32.7 ± 24.0	29.1 ± 12.6	20.9 ± 15.6						
	Shank-phase	Pre-training	51.4 ± 24.0	42.2 ± 19.1	53.1 ± 19.8	0.197	0.106	0.371	0.066	**0.003**	0.268
		Post-training	54.0 ± 27.1	58.1 ± 15.3	69.9 ± 18.1						
	Foot-phase	Pre-training	8.9 ± 4.0	9.6 ± 5.3	6.6 ± 2.8	0.587	0.036	0.235	0.095	0.277	0.041
		Post-training	8.9 ± 4.8	7.9 ± 3.9	6.1 ± 4.0						

The bold in *p*-Value indicates a significant difference (*p* < 0.05).

**Table 7:** Comparison of the time spent percentage of each coordination pattern in the leg acceleration phase between training groups and time.

Segment	Patterns	Time	Training Group			Time × Group Effect		Group Effect		Time Effect	
			Coordination (*n* = 12)	Strength (*n* = 10)	Kicking (*n* = 10)	*p* Value	η^2^p	*p* Value	η^2^p	*p* Value	η^2^p
Hip-knee	In- phase	Pre-training	39.5 ± 13.0	38.7 ± 10.2	37.9 ± 14.3	0.900	0.007	0.813	0.014	0.491	0.016
		Post-training	39.2 ± 8.7	36.7 ± 17.1	35.0 ± 11.2						
	Anti-phase	Pre-training	0.0 ± 0.0	0.1 ± 0.4	0.5 ± 1.2	0.187	0.109	0.463	0.052	0.690	0.006
		Post-training	0.0 ± 0.0	1.0 ± 3.2	0.0 ± 0.0						
	Thigh-phase	Pre-training	8.4 ± 7.4	6.2 ± 4.5	4.3 ± 3.1	0.555	0.040	0.113	0.139	0.526	0.014
		Post-training	6.9 ± 3.1	4.6 ± 3.3	5.3 ± 2.4						
	Shank-phase	Pre-training	52.2 ± 16.9	55.1 ± 13.4	57.3 ± 17.0	0.992	0.001	0.570	0.038	0.465	0.019
		Post-training	53.9 ± 10.3	57.7 ± 18.1	59.7 ± 12.4						
Knee-ankle	In- phase	Pre-training	17.4 ± 11.5	16.6 ± 12.2	20.6 ± 13.2	0.859	0.010	0.701	0.024	0.164	0.066
		Post-training	22.8 ± 12.5	19.4 ± 11.0	23.0 ± 12.6						
	Anti-phase	Pre-training	6.3 ± 5.7	7.1 ± 12.5	11.9 ± 7.6	0.233	0.096	0.202	0.104	0.051	0.125
		Post-training	9.0 ± 11.6	18.8 ± 12.7	13.3 ± 11.8						
	Shank-phase	Pre-training	69.1 ± 21.9	74.6 ± 20.1	67.2 ± 13.2	0.513	0.045	0.850	0.011	0.162	0.066
		Post-training	67.4 ± 16.2	61.4 ± 13.7	63.6 ± 16.8						
	Foot-phase	Pre-training	1.2 ± 3.3	1.7 ± 3.4	0.3 ± 0.4	0.694	0.025	0.335	0.073	0.291	0.038
		Post-training	0.8 ± 1.5	0.4 ± 1.3	0.2 ± 0.3						

**Table 8:** Comparison of the average relative activity between agonist and antagonist muscles in the kicking movement between training groups and time.

Muscles	Time	Training Group			Time × Group Effect		Group Effect		Time Effect	
		Coordination (*n* = 12)	Strength (*n* = 10)	Kicking (*n* = 10)	*p* Value	η^2^p	*p* Value	η^2^p	*p* Value	η^2^p
Quadriceps femoris–hamstrings	Pre-training	4.63 ± 3.65	7.71 ± 6.45	5.81 ± 6.20	0.545	0.046	0.743	0.023	0.142	0.081
	Post-training	4.17 ± 3.04	3.52 ± 4.59	4.39 ± 3.36						
Tibialis anterior– gastrocnemius	Pre-training	2.75 ± 1.49	1.81 ± 1.44	2.72 ± 1.25	**0.047**	0.218	**0.033**	0.238	0.401	0.028
	Post-training	2.00 ± 0.98 ^B^	2.15 ± 0.92 ^B^	4.36 ± 2.83 ^A^						

^A^ indicates statistical significance compared to the strength training group. ^B^ indicates statistical significance compared to the kicking training group. The bold in *p*-Value indicates a significant difference (*p* < 0.05).

## Data Availability

Data supporting the results presented in the manuscript are included in the figures and online resources whenever possible and are available upon request to the corresponding author.

## References

[1] Zhang L., Zhang M., Liu H. (2025). Inter-Segmental Coordination During Soccer Instep Kicking: A Vector-Coding Comparison Between Experienced Athletes and Novices. Bioengineering.

[2] Zhou Z., Gao Z., Li F., Wang D., Wang Y., Fekete G., Gu Y. (2025). Comparison of interlimb coordination during soccer instep kicking between elite and amateur players. Eur. J. Sport Sci..

[3] Bemstein N. (1967). The Co-Ordination and Regulation of Movements.

[4] Chang R., Van Emmerik R., Hamill J. (2008). Quantifying rearfoot–forefoot coordination in human walking. J. Biomech..

[5] Aagaard P., Simonsen E.B., Andersen J.L., Magnusson S.P., Bojsen-Møller F., Dyhre-Poulsen P. (2000). Antagonist muscle coactivation during isokinetic knee extension. Scand. J. Med. Sci. Sports.

[6] Gittoes M.J., Wilson C. (2010). Intralimb joint coordination patterns of the lower extremity in maximal velocity phase sprint running. J. Appl. Biomech..

[7] Anderson D.L., Sidaway B. (1994). Coordination changes associated with practice of a soccer kick. Res. Q. Exerc. Sport.

[8] Li Y., Alexander M., Glazebrook C., Leiter J. (2016). Quantifying inter-segmental coordination during the instep soccer kicks. Int. J. Exerc. Sci..

[9] Chow J.Y., Davids K., Button C., Koh M. (2007). Variation in coordination of a discrete multiarticular action as a function of skill level. J. Mot. Behav..

[10] Seifert L., Leblanc H., Herault R., Komar J., Button C., Chollet D. (2011). Inter-individual variability in the upper–lower limb breaststroke coordination. Hum. Mov. Sci..

[11] Seifert L., Leblanc H., Chollet D., Delignières D. (2010). Inter-limb coordination in swimming: Effect of speed and skill level. Hum. Mov. Sci..

[12] Seifert L., Wattebled L., L’Hermette M., Herault R. (2011). Inter-limb coordination variability in ice climbers of different skill level. Balt. J. Sport Health Sci..

[13] Chardonnens J., Favre J., Cuendet F., Gremion G., Aminian K. (2013). Characterization of lower-limbs inter-segment coordination during the take-off extension in ski jumping. Hum. Mov. Sci..

[14] Chow J.Y., Davids K., Button C., Koh M. (2008). Coordination changes in a discrete multi-articular action as a function of practice. Acta Psychol..

[15] Egan C.D., Verheul M.H., Savelsbergh G.J. (2007). Effects of experience on the coordination of internally and externally timed soccer kicks. J. Mot. Behav..

[16] Kaufman L.B., Schilling D.L. (2007). Implementation of a strength training program for a 5-year-old child with poor body awareness and developmental coordination disorder. Phys. Ther..

[17] Rutherford O. (1988). Muscular coordination and strength training. Sports Med..

[18] Rutherford O., Jones D. (1986). The role of learning and coordination in strength training. Eur. J. Appl. Physiol. Occup. Physiol..

[19] NSCA (2019). Developing Agility and Quickness.

[20] Cortis C., Tessitore A., Perroni F., Lupo C., Pesce C., Ammendolia A., Capranica L. (2009). Interlimb coordination, strength, and power in soccer players across the lifespan. J. Strength Cond. Res..

[21] Jullien H., Bisch C., Largouët N., Manouvrier C., Carling C.J., Amiard V. (2008). Does a short period of lower limb strength training improve performance in field-based tests of running and agility in young professional soccer players?. J. Strength Cond. Res..

[22] Wan X., Li S., Best T.M., Liu H., Li H., Yu B. (2021). Effects of flexibility and strength training on peak hamstring musculotendinous strains during sprinting. J. Sport Health Sci..

[23] Hasan S. (2023). Effects of plyometric vs. strength training on strength, sprint, and functional performance in soccer players: A randomized controlled trial. Sci. Rep..

[24] Rodriguez-Lorenzo L., Fernandez-del-Olmo M., Martin-Acero R. (2016). Strength and Kicking Performance in Soccer: A Review. Strength Cond. J..

[25] Manolopoulos E., Katis A., Manolopoulos K., Kalapotharakos V., Kellis E. (2013). Effects of a 10-week resistance exercise program on soccer kick biomechanics and muscle strength. J. Strength Cond. Res..

[26] Yu B., Gabriel D., Noble L., An K.-N. (1999). Estimate of the optimum cutoff frequency for the Butterworth low-pass digital filter. J. Appl. Biomech..

[27] Brophy R.H., Backus S., Kraszewski A.P., Steele B.C., Ma Y., Osei D., Williams R.J. (2010). Differences between sexes in lower extremity alignment and muscle activation during soccer kick. J. Bone Jt. Surg..

[28] Robertson D., Caldwell G., Hamill J., Kamen G., Whittlesey S. (2013). Research Methods in Biomechanics.

[29] Hamill J., Haddad J.M., McDermott W.J. (2000). Issues in quantifying variability from a dynamical systems perspective. J. Appl. Biomech..

[30] Zhang L., Li H., Garrett W.E., Liu H., Yu B. (2020). Hamstring muscle-tendon unit lengthening and activation in instep and cut-off kicking. J. Biomech..

[31] Konrad P. (2005). The ABC of EMG. A Practical Introduction to Kinesiological Electromyography.

[32] Scurr J., Hall B. (2009). The effects of approach angle on penalty kicking accuracy and kick kinematics with recreational soccer players. J. Sport. Sci. Med..

[33] Shan G., Westerhoff P. (2005). Soccer: Full-body kinematic characteristics of the maximal instep soccer kick by male soccer players and parameters related to kick quality. Sports Biomech..

[34] Afonso J., da Costa I.T., Camões M., Silva A., Lima R.F., Milheiro A., Martins A., Laporta L., Nakamura F.Y., Clemente F.M. (2020). The Effects of Agility Ladders on Performance: A Systematic Review. Int. J. Sports Med..

[35] Vereijken B., van Emmerik R.E., Whiting H., Newell K.M. (1992). Free (z) ing degrees of freedom in skill acquisition. J. Mot. Behav..

[36] Temprado J., Della-Grasta M., Farrell M., Laurent M. (1997). A novice-expert comparison of (intra-limb) coordination subserving the volleyball serve. Hum. Mov. Sci..

[37] Swinnen S.P., Jardin K., Meulenbroek R., Dounskaia N., Den Brandt M.H.V. (1997). Egocentric and allocentric constraints in the expression of patterns of interlimb coordination. J. Cogn. Neurosci..

[38] Jirsa V.K., Friedrich R., Haken H., Kelso J.S. (1994). A theoretical model of phase transitions in the human brain. Biol. Cybern..

